# Contact‐Free Human‐Machine Interfaces via Printed Thermopile Infrared Arrays

**DOI:** 10.1002/advs.78569

**Published:** 2026-10-29

**Authors:** Zirui Wang, Nan Luo, Amrutha Bose, Muhammad Irfan Khan, Jiayi Liu, Leon Viktor Klinder, Leonard Franke, Mohamed Hussein, Uli Lemmer, Md Mofasser Mallick

**Affiliations:** ^1^ Light Technology Institute Karlsruhe Institute of Technology (KIT) Karlsruhe Germany; ^2^ Institute of Microstructure Technology Karlsruhe Institute of Technology (KIT) Eggenstein‐Leopoldshafen Germany; ^3^ varmo GmbH Eggenstein‐Leopoldshafen Germany; ^4^ Department of Physics Faculty of Science Ain Shams University Abbassia Cairo Egypt

**Keywords:** Bi_2_Te_3_, human‐machine‐interaction, infrared sensing, printed thermoelectrics, Seebeck effect

## Abstract

Human‐machine interfaces (HMIs) are evolving as key components for advanced sensing in communication and control systems. In particular, contact‐free HMIs can facilitate unique advantages in hygiene, durability, and non‐destructive sensing. However, spatially resolved sensing platforms remain challenging using bulk approaches. Here, we report flexible infrared interface arrays (TIA1 and TIA2) with ≥10 pixels that exploit the photo‐thermoelectric (PTE) infrared sensing properties of Bi‐Te‐based thermoelectric (TE) materials via a dual‐stage energy conversion process: radiation‐to‐thermal, followed by thermal‐to‐electric. The p‐ and n‐type TE materials and electrodes were printed on polyimide to fabricate TIAs. After encapsulation with a black absorber, radially patterned silver mirrors are printed on the outer part of the pixels using aerosol jet‐printing to partially shield each thermocouple. This induces a localized temperature gradient, hence an output voltage via the Seebeck effect under infrared illumination. The pixels exhibit responsivities of 0.7 VW^−1^ (TIA1) and 0.9 VW^−1^ (TIA2), respectively, under CO_2_ laser irradiation (10.6 µm), with pixel‐to‐pixel variation within ± 10%. The TIAs were demonstrated as non‐contact handwriting and dialing interfaces, converting human gestures into digital signals. These results pave the way for a self‐powered and contactless interface for future human–machine interaction technologies.

## Graphical abstract

Can a printed sheet locate and sense a fingertip without being touched? Fully printed, flexible thermoelectric arrays convert the infrared from a hovering finger directly into voltage, with no external power. Aerosol‐jet‐printed silver mirrors partially shield each pixel, creating a local temperature gradient. With responsivities up to 0.9 V W^−^^1^, the arrays enable contact‐free dialing and handwriting.

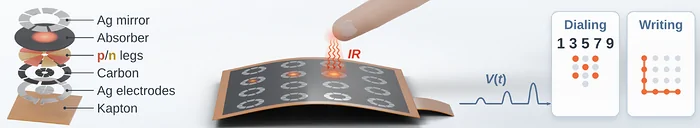

## 1 Introduction

Research on thermoelectrics has largely focused on applications in energy harvesting, waste‐heat recovery, and solid‐state cooling. These applications rely on the conversion of heat into electrical energy through the Seebeck effect and the reverse process, electrical energy to heat, through the Peltier effect. Consequently, most advances in thermoelectric (TE) materials and devices have focused on these two applications: thermoelectric generators (TEGs) for energy harvesting and waste heat recovery [1, 2, 3, 4, 5, 6, 7, 8, 9], and thermoelectric coolers (TECs) for solid‐state cooling [10, 11, 12, 13, 14]. Beyond these two applications, TE technology is also employed for thermal sensing. Despite this area receiving less attention, TE sensors remain highly relevant for sensing applications, including temperature [15, 16, 17, 18, 19, 20], heat flux [21, 22, 23, 24, 25], and radiation sensing [26, 27, 28]. TE thermal sensors are governed by the Seebeck voltage generated by a localized temperature rise at one junction of a thermocouple (TC) due to heat absorption via conduction, convection, or radiation. Among all TE thermal sensors, temperature sensors have found the broadest commercial adaptation. However, with growing demands in contactless infrared radiation sensing and imaging, TE infrared sensors can play a vital role owing to their ability to detect broadband infrared via the photothermoelectric (PTE) effect. The working mechanism of these infrared sensors involves a dual‐stage energy conversion process: radiation‐to‐thermal, followed by thermal‐to‐electric via the Seebeck effect. The absorbed radiation energy *Q* induces a temperature rise (Δ*T*) at the hot junction of the sensors, which can be described as:

(1) $$\Delta T=\frac{Q}{mc_{p}}\;$$

where *m* is the mass of the heated volume and *c*_p_ is its specific heat capacity. The corresponding thermoelectric voltage per thermocouple generated by the temperature difference Δ*T* resulting from the Seebeck effect is:

(2) $$V=\left(\alpha_{p}-\alpha_{n}\right)\;\Delta T=\left(\alpha_{p}-\alpha_{n}\right)\frac{Q}{mC_{p}}$$

where *α*_p_ and *α*_n_ represent the Seebeck coefficients of the p‐type and n‐type legs, respectively. In the case of serially connected thermocouples (thermopile), the voltage has to be multiplied by N, with N being the number of thermocouples in the PTE infrared sensor. Hence, PTE infrared sensors directly convert the absorbed radiation energy into electrical signals, making them promising candidates for detecting radiation [26, 29, 30], including thermal radiation spectrally located in the infrared (IR) [31, 32, 33]. Beyond the PTE effect, photothermal conversion can facilitate IR detection through heat‐induced changes in electrical resistance, as demonstrated by the photobolometric response of Bi_2_Se_3_/MoTe_2_ heterostructures [34]. Defect‐assisted photogating offers another detection mechanism in quasi‐1D PtSe_2_ [35]. IR detection is vital for numerous applications, such as security, motion detection, proximity sensing, and human‐machine interaction (HMI) [27, 28, 36, 37]. Presently, IR detectors mainly utilize semiconductor materials with a narrow bandgap [38, 39], such as mercury cadmium telluride (MCT), which relies on electron‐hole generation across the semiconductor bandgap. However, these detectors require external bias and cryogenic cooling, which makes them costly and complex. In contrast, PTE IR sensors can be self‐powered and operated at room temperature. However, the traditional PTE IR sensors are fabricated via physical vapor deposition (PVD), followed by lithography. These fabrication approaches are not low‐cost and do not enable mechanical flexibility and shape‐conformability. In recent years, several studies have attempted to fabricate PTE sensors using wet‐processed TE films [40], such as those deposited by doctor‐blading, drop casting, and printing, to address these limitations. Printing has emerged as a versatile approach for thermoelectrics [36, 41], enabling precise patterning of TE materials on a wide range of flexible and non‐flexible substrates to fabricate low‐cost, shape‐conformable TE devices [42]. Hence, integrating high‐throughput printing technologies like screen‐printing is also an attractive method for further facilitating low‐cost, scalable manufacturing of flexible PTE infrared sensors.

With the rapid advancement of communication systems in the Internet of Things (IoT) [43, 44], smart home [45], and virtual/augmented reality [46, 47], HMI systems are attracting growing interest for their potential to serve as interfaces between humans and machines [27, 28, 37, 46, 48]. These systems can receive information from users, process and translate it into commands to operate electronic systems. However, most of those HMI systems are based on physical contact between the human and the device by inducing an electric signal based on capacitance [49], friction [50, 51], pressure [44], pyroelectric [52], or conductive temperature sensors [16, 18, 19]. This leads to soiling and mechanical abrasion after long‐term use, leading to maintenance and replacement costs. Moreover, the COVID pandemic has further highlighted how public contact‐based devices can transmit the virus and cause cross‐infection [53, 54]. Therefore, contact‐free HMI systems based on different mechanisms are explored, such as voice [55, 56], humidity [57], and optical motion tracking [58, 59]. More recently, a combination of thermoelectric and triboelectric HMI was demonstrated [60]. HMI systems have thus become more common, though most are still affected by environmental noise and may lack precision. PTE infrared sensors and HMIs [27, 31, 37, 61], by contrast, can offer a self‐powered, robust, and movement‐free non‐contact communication method. A comparison table among different HMI systems is shown in Table S1. However, most of the non‐contact HMI systems mentioned above feature rigid structures, often involve high cost, and significant power consumption. Thus, flexible, large‐area, and self‐powered HMI systems enabled by *printed* TE materials have the potential to be transformative.

In this work, we report fully printed p‐ and n‐type Bi‐Te‐based flexible thermoelectric infrared interface arrays (TIAs). The interface arrays can locate thermal stimuli via the photo‐thermoelectric (PTE) infrared sensing properties of the printed Bi‐Te‐based thermocouples. The TIAs were fabricated using screen printing on flexible substrates. A localized temperature gradient was created under infrared illumination by partially shielding the thermocouples in the TIA pixels using an aerosol‐jet printed highly reflective silver mirror. As a result, an output voltage is generated via the Seebeck effect (cf. Figure 1). The TIA pixels exhibit responsivities of up to 0.9 VW^−1^. The TIAs can detect hand and finger movements by sensing the thermal signature. Finally, they are demonstrated as non‐contact handwriting and dialing interfaces using a readout circuit and a graphical user interface (GUI). These results indicate the potential of the printed TE infrared interface as low‐cost and self‐powered contact‐free human–machine interaction systems.

**FIGURE 1:**
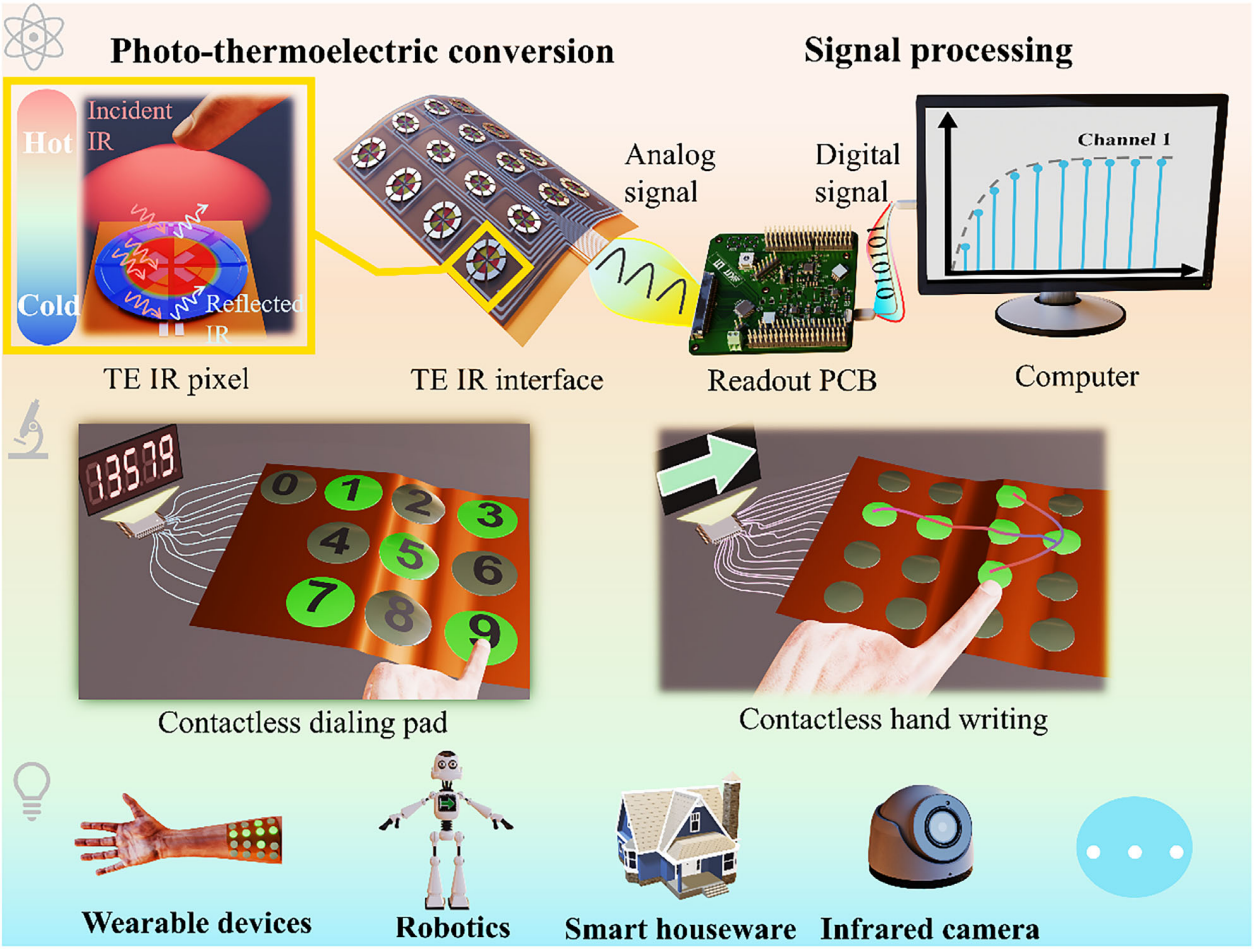
Fundamentals, experimental demonstration, and prospective application of printed flexible thermoelectric infrared interfaces.

## 2 Experimental Methods

### 2.1 Materials

Ingots of p‐type Bi_0.5_Sb_1.5_Te_3_ and n‐type Bi_2_Te_2.7_Se_0.3_ (ingots, 99%, EVERREDtronics), Copper powder (spheroidal) (10–25 µm, 98%, Sigma–Aldrich), Selenide powder (100 mesh, ≥ 99.5% trace metals basis, Sigma–Aldrich), Polyvinylpyrrolidone (PVP) (average Mw ≈ 40 000, Sigma–Aldrich), N, N‐Diethylformamide (DEF) (99%, Sigma‐Aldrich), terpineol (α‐Terpineol, 97%, Sigma Aldrich), silver ink (Thermo Fischer), carbon ink (Dycotec, DM‐CAP‐4701S), and Kapton substrate (25 µm thickness, Dietrich Müller GmbH), JS‐A221AE SILVER Nanoparticle aerosol ink (50 wt.%, Novacentrix).

### 2.2 Preparation of p‐Type and n‐Type TE Inks

The preparation of the inks followed the previously reported method [15, 62]. The printable TE inks were prepared by wet milling of inorganic particles (TE particles and Cu_2_Se binder) and an organic solution. The TE particles used in the p‐type and n‐type TE inks were p‐type Bi_0.5_Sb_1.5_Te_3_ (p‐BST) and n‐type Bi_2_Te_2.7_Se_0.3_ (n‐BT), respectively. The inorganic binder (IB) was composed of Cu and Se powders in the stoichiometric ratio of 2:1. The IB accounted for 10 wt.% and 5 wt.% of the total solid loading in the p‐type and the n‐type inks, respectively. The organic solution was prepared by dissolving 8 wt. % of the organic binder, PVP, in a blend of solvents. The solvent blend consisted of terpineol and DEF in a 4:1 ratio. The ground p‐BST and n‐BT TE powders were blended with the IB and the organic solution at a weight ratio of 3:1 (inorganic particles to organic solution). Then, the mixtures were sealed in zirconia jars. After a gentle nitrogen (N_2_) purge for 5 min, the final mixtures were ball‐milled using a Fritsch Planetary Mill (PULVERISETTE 5 premium line) at 300 rpm for 60 min. The resultant inks were stored under inert conditions for subsequent use in printing. The measured average dynamic viscosity of the p‐BST ink was 431.5 mPa·s, whereas that of the n‐BT ink was 845.2 mPa·s, as shown in Table S2.

### 2.3 Fabrication of Fully Printed TE Infrared Interface Arrays (TIAs)

Two layouts of printed TE infrared interface arrays (TIAs) were fabricated using p‐BST and n‐BT TE inks, comprising 10 (TIA1) and 16 (TIA2) active thermopile sensing pixel elements, respectively. The TIAs were screen‐printed on a flexible Kapton substrate with a thickness of 25 µm, chosen for its excellent thermal stability, electrical insulation, and mechanical flexibility. Screen printing was employed throughout the process due to its capability to produce uniform and precisely patterned films suitable for scalable, high‐quality fabrication. The fabrication process began with screen printing the silver paste to form electrodes on the Kapton substrate (cf. Figure 2a). These electrodes were then annealed on a hot plate at 423 K to ensure low resistance. Following this, a carbon paste was screen‐printed as an interfacial layer to reduce the contact resistance between the metallic electrodes and the TE legs. Next, the p‐BST and the n‐BT inks were printed onto predefined electrodes. The printed TE legs were subsequently thermally sintered on a hot plate inside a glovebox at 623 K for 10 min. The fabrication routine for the TIAs is shown in Figure 2a, and photographs of the different layers are shown in Figure S1. The overall sensor array dimensions were 118 mm × 100 mm (TIA1) and 115 mm × 90 mm (TIA2), respectively. Each radial pixel consists of 4 thermocouples, and the pixel radii were 10 mm (TIA1) and 7.5 mm (TIA2), with spacings between surrounding pixels of 31 mm (TIA1) and 26 mm (TIA2), respectively. These sufficient spacings for both configurations ensure good spatial and temperature resolution (cf. Note S1). The thicknesses of the printed TE films were measured to be around 22 µm. To improve interlayer adhesion and reduce interfacial resistance, the device was hot‐pressed, then encapsulated with a carbon absorber tape to enhance mechanical robustness and infrared absorption. As the last step, a silver mirror with high reflectivity in the IR range was fabricated using Aerosol Jet printing (Optomec 5X). The silver mirror radial segments were deposited on the outer part of the TE leg with radial dimensions of 4.5 mm (TIA1) and 3 mm (TIA2), including 1 mm offsets for each pixel to ensure better filling quality. The aerosol jet‐printed (AJP) silver provides a smooth surface quality, which can serve as an excellent reflector for IR. This in‐plane layout of the TE sensing pixel (cf. Figure 2b), with one absorber and one reflector area each, maintains the temperature gradient within the leg, enabling it to respond not only to concentrated IR sources but also to larger‐area IR sources. The details on screen printing precision are discussed and shown in Figure S2.

**FIGURE 2:**
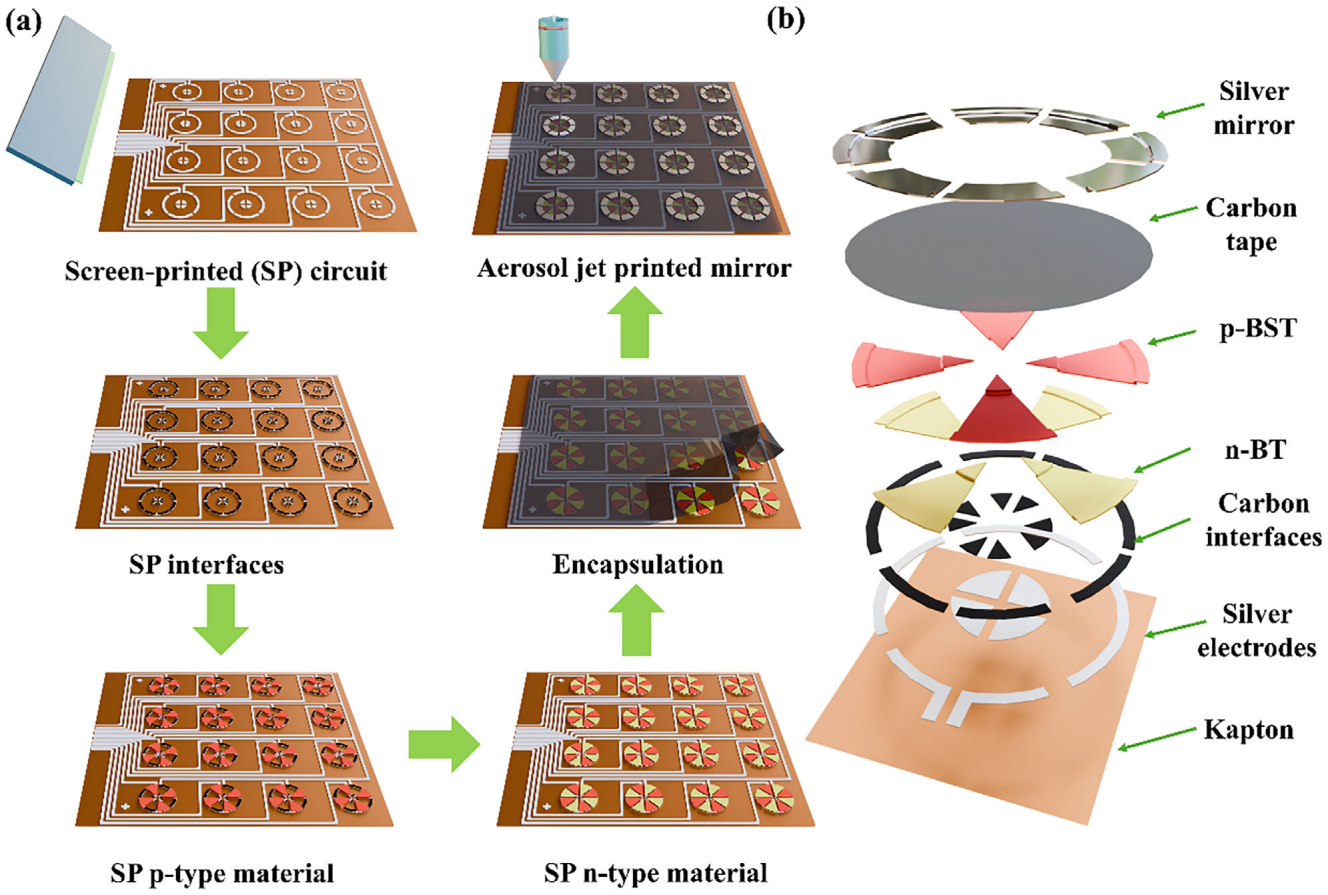
(a) Fabrication process of printed TIAs (here TIA2) using a combination of two printing techniques. (b) A schematic of the stacked structure, showing the individual functional printed layers of the pixel.

### 2.4 Characterization Techniques of Printed TE Films and TIAs

Different characterization techniques were employed to get insights into the structural, morphological, and functional properties of the printed TE films and TIAs. A Bruker Vertex 80 FTIR spectrometer equipped with a Spectra Tech Diffuse Reflectance Infrared Fourier Transform Spectroscopy (DRIFTS) accessory was used to analyze the absorption properties of the TE films in the IR. A JEOL CarryScope JCM‐5700 Scanning Electron Microscope (SEM) was used to analyze the surface morphology. Bruker Contour GTK White Light Interferometry (WLI) was used to further assess the surface topography of the printed films. To ensure flawless printability and consistent film formation, the formulated inks’ rheological characteristics were examined using the Anton Paar ViscoQC 300 Viscometer. A Linseis HCS 10 system was used to measure the electrical conductivity (*σ*) and the Seebeck coefficient (*α*) to assess the TE performance. A Coherent Diamond C‐20 CO_2_ laser with a wavelength of 10.6 µm was used to study the PTE response under infrared laser irradiation. Moreover, to visualize the performance of the TIAs, an electronic readout circuit with analog signal processing was integrated on a printed circuit board (PCB). A 32‐channel FPC zero‐insertion‐force connector was used to input the signals from the array. Following this, a multiplexer ADG731 enables scalable data selection and acquisition. INA333 instrument amplifiers were used to build differential stages that amplify the input signals. Then, ADS1220, a 24‐bit resolution analog‐to‐digital converter (ADC), was connected to digitize the signal and transfer the data to the host, where we used the STM32 Nucleo‐64 as the host controller interface.

## 3 Experimental Results

### 3.1 Microstructural and Morphological Properties of the Printed TE Films

Figure 3a,b presents the microstructures and morphologies of the printed p‐ and n‐type TE films, respectively. The SEM images show particle distributions in both p‐BST and n‐BT TE films, even though the macroscopic views present homogeneous printed films. The microscopic images also reveal that both p‐BST and n‐BT films comprise particles ranging from 1 to 5 µm. High viscosities help prevent ink spreading and ensure excellent printing accuracy (cf. Figure 3e). The microstructures of the aerosol‐jet‐printed silver reflectors are shown in Figure 3c,d. The surface is found to be uniform and compact due to silver nanoparticles in the ink, which enhance its reflectivity. However, some voids and porosity (cf. Figure 3c–d) were formed during printing the reflector due to a non‐uniform surface (carbon encapsulation). Despite those defects, the AJP silver reflectors exhibit a high infrared reflectivity because the nanovoids only slightly affect the diffuse reflection of the long‐wave infrared (LWIR) wavelength. More SEM images are shown in Figure S3. Figure 3e shows the height distribution of the screen‐printed TIA pixels (here TIA2; TIA1 is presented in Figure S4) along both the x‐ and y‐axes, as well as the 3D reconstructed topography map using a WLI. Due to its mechanical flexibility, the substrate could not be properly flattened on the interferometer stage. Therefore, the total height in a whole pixel area ranges from 27 µm to 151 µm. A small area of the printed thermocouple leg was magnified, and the 3D roughness parameter (*S*_a_) was measured at 1.168 µm, indicating excellent uniformity across the whole device. This indicates high smoothness of TE films obtained by screen printing.

**FIGURE 3:**
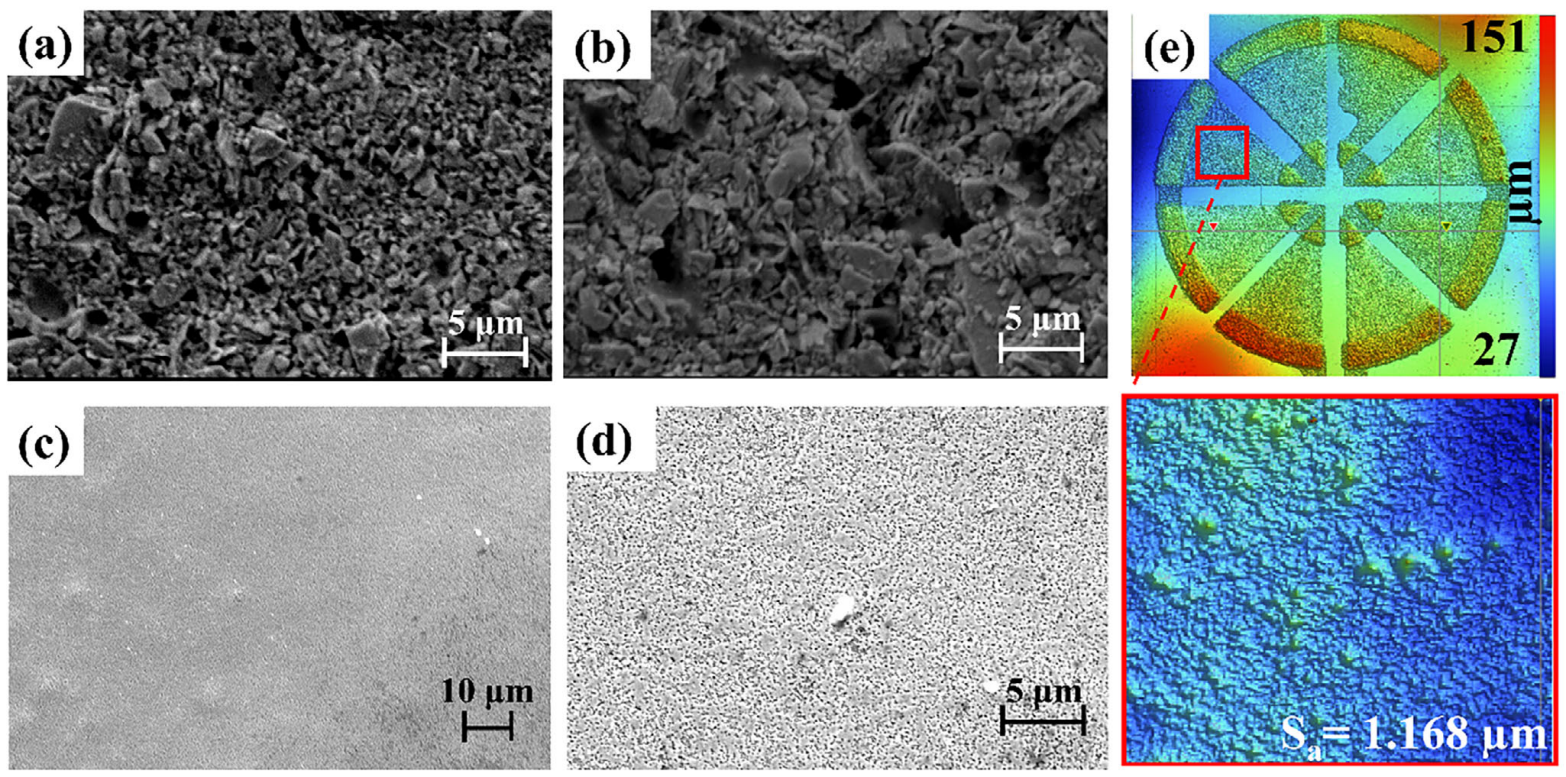
SEM and WLI analysis of printed TE films and silver mirror. (a) SEM image of printed p‐BST films. (b) SEM image of printed n‐BT films. (c) and (d) depict SEM images of printed silver mirror on the pixel at different magnifications. (e) 2D roughness distribution (upper) and zoomed view of screen‐printed TE film (lower).

### 3.2 Mechanical Properties of the Printed TE Films and TIAs

The mechanical flexibility of the printed TIAs is important for applications on non‐planar surfaces. To study the mechanical flexibility in detail, the printed TE films were first subjected to bending tests for 100 cycles with a 10 mm bending diameter (cf. Figure 4a). The changes in normalized resistances of p‐ and n‐type TE films with bending cycles are presented in Figure 4b). After 20 bending cycles, the resistances of p‐ and n‐type films increased by 14.5% and 35%, respectively. From 20 to 100 cycles, the normalized resistances of both films varied only slowly and reached 1.17 and 1.46, indicating good mechanical robustness. Using the same bending diameter, we again tested the two TIAs up to 100 bending cycles (cf. Figure 4c). A single pixel from each TIA was selected, and its resistance change was tracked. The changes in normalized resistance follow those of the TE films, with only moderate changes in resistance observed after 100 bending cycles. Additionally, we recorded the average normalized resistances of TIA1 and TIA2, before and after 100 bending cycles. The results show that, despite slight differences in initial resistance, all the pixels underwent similar changes after 100 cycles, suggesting good reproducibility for both the TE films and the silver electrode. The good mechanical robustness and flexibility of the arrays highlight the potential for wearables and large‐area fabrication. Further long‐term and bending stability tests of TIAs are presented in Note S2.

**FIGURE 4:**
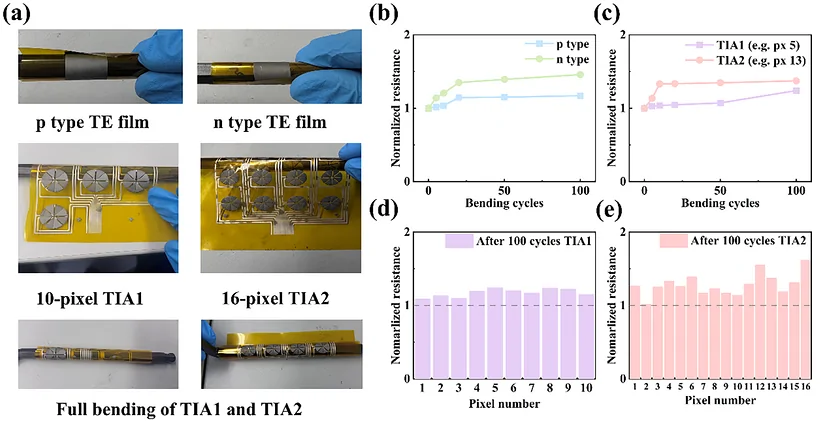
Mechanical properties of printed TE films and the TIAs. (a) Bent TE films and TIAs with a 10 mm diameter cylinder. (b) Normalized resistances of TE films vs. number of bending cycles. (c) Normalized resistances of a random pixel vs. number of bending cycles. Averaged normalized resistance after 100 bending cycles (d) TIA1 and (e) TIA2.

### 3.3 Optical and Thermoelectric Properties of Printed n‐ and p‐Type Films

The performance of the TIAs is influenced by the individual pixel's ability to convert infrared radiation into heat. Therefore, the optical properties of the printed TE films, the encapsulated absorber, and the printed silver mirror were studied. First, the long‐wave infrared (8–14 µm) absorption properties of the 22 µm thick printed TE films on a polyimide substrate were investigated using an FTIR spectrometer. Both TE films exhibit high absorption, typically between 65% and 80%, across the entire wavelength range (cf. Figure 5a). This proves that both types of TE films have strong absorption in the LWIR region. Since the TIAs were encapsulated with a black absorber, it is necessary to check the absorptance of the encapsulated films, as shown in Figure 5b. The film absorptance (blue curve) was improved to around 90%. The transmissivity, shown as a purple curve, was measured to be from 0% to 20%. This indicates that the encapsulation tape facilitates the absorption of LWIR radiation, with nearly no light reflected from the absorber region. In addition, the AJP silver mirror in the outer area of the pixel was measured in reflection mode (cf. Figure 5c). The reflectance of the silver mirror deposited directly on the Kapton substrate was near unity and was still above 90% over the whole wavelength range when the silver was deposited on the TE‐pixel. These findings indicate a good functional printed reflector for LWIR radiation. These three synergistic optical spectra demonstrate the needed optical contrast to create a temperature difference between the two junctions of the thermocouples in a pixel.

**FIGURE 5:**
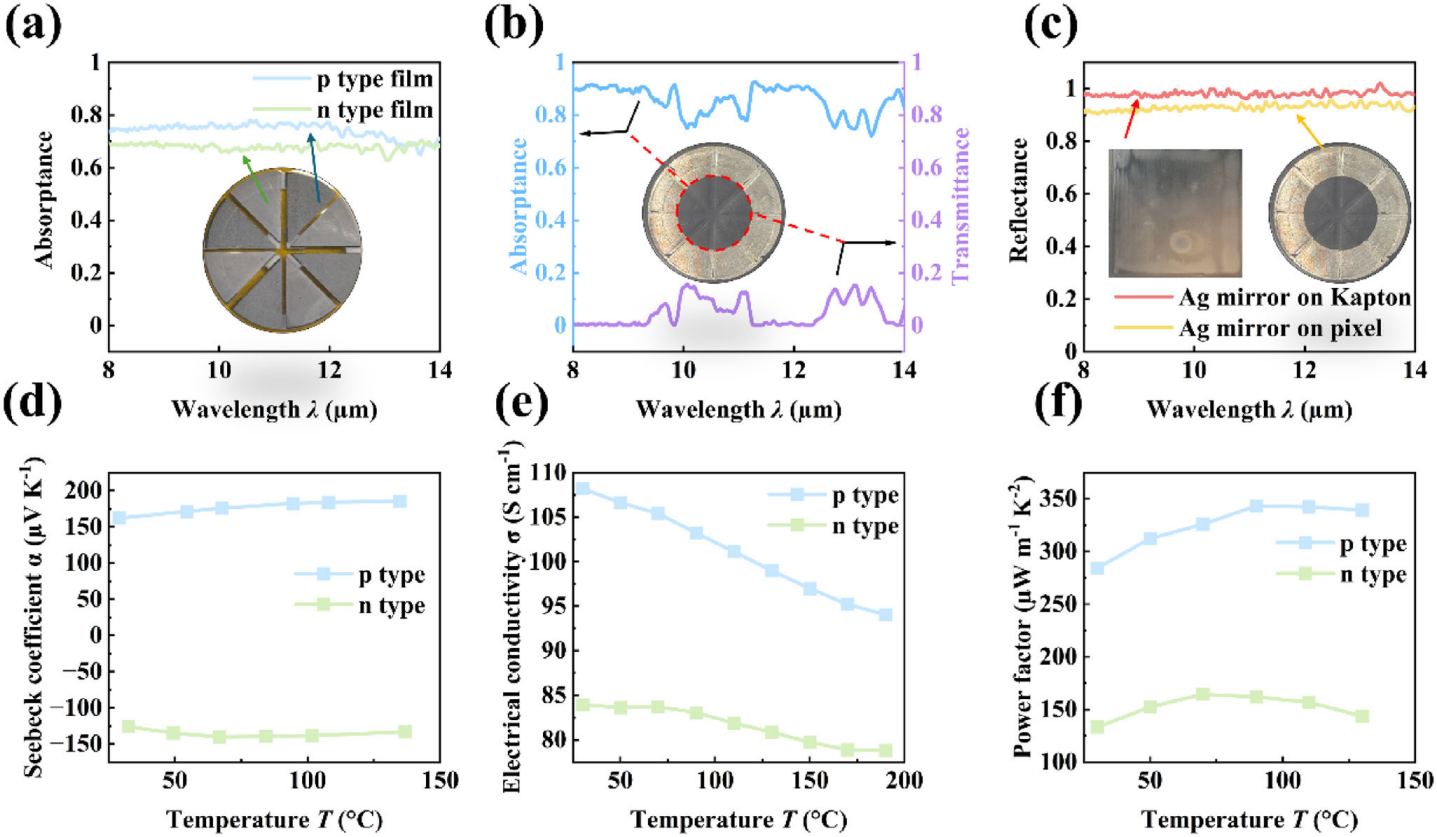
Optical (LWIR wavelength) and thermoelectric properties of n‐ and p‐type printed films. (a) Absorptance of p‐ and n‐type films. (b) Absorptance and transmittance of the encapsulated absorber. (c) Reflectances of the aerosol‐jet printed silver mirror on Kapton and on the pixels. The temperature‐dependent (d) Seebeck coefficients *α*. (e) Electrical conductivities *σ*. (f) Power factors.

The PTE performance is governed by the local absorption of radiation and the material's ability to convert the temperature gradient to an electrical signal via the Seebeck effect. Therefore, the Seebeck coefficient was measured to assess the printed films’ PTE performance. At room temperature, the Seebeck coefficient (α) for the p‐type film was 162 µVK^−1^, whereas for the n‐type film it was ‐126 µVK^−1^ (cf. Figure 5d). The polarity of the Seebeck coefficients confirms the nature (p‐type vs. n‐type) of the BST and BT materials, respectively. The *α* is found to increase with temperature for both p‐ and n‐type films. The electrical conductivities (σ) for p‐ and n‐type were measured to be 108 and 84 Scm^−1^ at room temperature (cf. Figure 5e), indicating a good electronic functionality. The conductivities slightly decrease with temperature, indicating a decrease in carrier mobilities. The temperature‐dependent power factors for both films are shown in Figure 5f. They are found to be 280 and 120 µW m^−1^K^−2^ for p‐ and n‐type films, respectively.

### 3.4 Infrared Photo‐Thermoelectric (PTE) Performance of Individual Pixels of TIAs

#### 3.4.1 PTE Characterization of Pixels Using a CO_2_ Laser

After the investigation of optical and TE properties of the printed films, the overall PTE characterization was done using a CO_2_ laser as a well‐defined infrared source to determine four key parameters of the sensor pixels: Responsivity *R*, time constants *τ*_rise_ and *τ*_decay_, and dark noise voltage *V*_noise_. The responsivity *R* of the sensor pixel is defined by the ratio of output voltage *V*_out_ to the input optical power *P*_in_, expressed as *R*  =  *V*_out_/*P*_in_. The time constants show how fast a sensor pixel can react to thermal stimuli. They can be determined using the following exponential equations: $V_{\mathrm{out}}=V_{0}\;(1-e^{-\frac{t}{\tau_{\mathrm{rise}}}}$) during rise and $V_{\mathrm{out}}=V_{0}\;e^{-\frac{t}{\tau_{\mathrm{decay}}}}$ during decay, where *V*_0_ in both cases represents the steady‐state output that the pixel reaches under the respective input power. The noise voltage *V*_noise_ is defined as the root‐mean‐square (RMS) value of the pixel output without any thermal stimuli over a measuring bandwidth *Δ* *f*. *V*_noise_ mainly depends on the Johnson noise and pixel resistance (*R*_P_), which can be expressed as $V_{\mathrm{noise}}=\sqrt{4k_{B}TR_{P}\Delta f}$. Here, *k*_B_ is the Boltzmann constant and *T* is the absolute temperature. The detailed measurement of Johnson noise is shown and discussed in Note S3. Figure 6a illustrates the characterization setup for measuring the PTE performance of an individual pixel using a CO_2_ laser operating with a wavelength of 10.6 µm. The laser power is attenuated in two stages by zinc selenide (Zn/Se) beam splitters, and a commercial IR detector is used to quantify the laser power as a reference. The incident laser power on a single pixel was varied from 0 to 10 mW, with a spot size of around 4 mm, while maintaining a thermally stable operating environment for the CO_2_ laser. For the noise measurement, the single PTE pixel was connected to a nanovoltmeter with a 12 Hz measurement bandwidth, whose real‐time data can be transferred to and processed by a computer. First, the saturated output voltages *V*_out_ for different laser powers *P*_in_ for both TIA1 and TIA2 pixels were measured and recorded. The different *V*_out_ could be linearly fitted with *P*_in_, where the slopes of the fitted curves represent the responsivities of the pixels (cf. shown in Figure 6b,e). The pixel from TIA1 exhibits a responsivity of 0.7 V/W, and the pixel from TIA2 shows a responsivity of 0.9 V/W. This difference in responsivity can be attributed to the different dimensions of the pixels and the thermal healing length.

**FIGURE 6:**
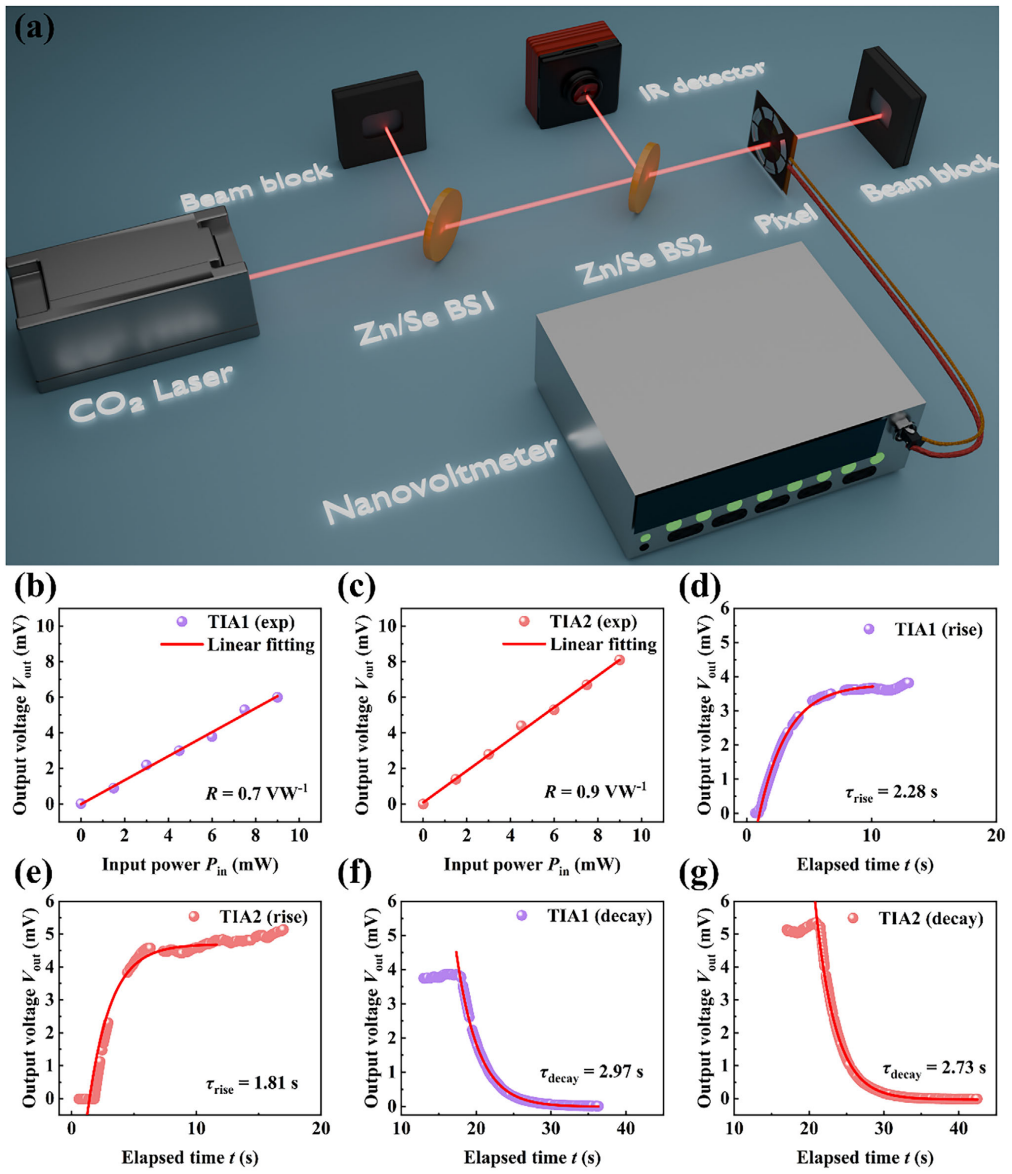
PTE characterization of individual pixels. (a) Illustration of the laser measurement setup. Variation of ***V***_**out**_ with different incident laser power ***P***_**in**_ for TIA1 (b) and TIA2 (c). Time‐dependent output voltage during rise after switching on the laser for TIA1 (d) and TIA2 (e). Time‐dependent output voltage during decay after switching off the laser for TIA1 (f) and TIA2 (g).

With zero input power, 1000 readings of *V*_out_ were recorded to assess the noise. For a pixel with 880 Ω internal resistance, the *V*_noise_ was estimated to be 18 nV, compared to its theoretical value for the Johnson noise of 14 nV at 303 K. The deviation may result from electrical background noise and radiation. After the stationary characterization, the dynamic performances of both types of pixels were investigated using *P*_in_ of 6 mW, as shown in Figure 6. The *V*_out_ was recorded as a function of elapsed time (*t*) to analyze the real‐time responses of the pixels. Both TIA1 and TIA2 pixels exhibit a response with time constants *τ*_rise‐TIA1_ and *τ*_rise‐TIA2_ of 2.28 s and 1.81 s (cf. Figure 6b,c), respectively. The difference in time constants is due to the larger leg in TIA1, which comprises higher thermal mass. Therefore, it requires more energy to reach the same temperature, resulting in a longer time to reach steady state. Regarding the decay time constants, without any external cooling for each pixel, the pixels from TIA1 and TIA2 featured 2.97 s and 2.73 s (cf. Figure 6e,f). For applications, it is not necessary to wait until the pixels have reached equilibrium; by electronically processing the output signal with a threshold current and fast channel switching, a sub‐second response is possible.

#### 3.4.2 Interaction of the Sensor Pixels With Human Infrared Source

A key factor to consider is how the IR source's position affects the pixel's signal. Hence, the response from a single pixel was studied by changing the relative finger position. Two different modes of position change were investigated: First, we examined the effect of a lateral offset (cf. Figure 7a), where a fingertip stays above the pixel at 5 lateral positions. Second, we tested the influence of a vertical offset (cf. Figure 7d), where the fingertip stays at 5 positions along a vertical straight line. The measurement details are shown in Note S4. The reference interaction position is where the finger's tip is just on top of the center of the pixel, with a distance of approximately 2 cm. Figure 7b,c,e,f shows the averaged response at 5 discrete positions over a 20s measurement period and their standard deviation (SD). The single pixel will show the highest output when the fingertip is nearest to the absorber part of the pixel. And due to the design with a large spacing (> 2 cm) between pixels, the correct activation of a pixel will not affect the neighboring pixel. Unlike the laser characterization discussed in the last section, the human finger can be approximated as a point source emitting a spherical wave. Given that IR radiation cannot be concentrated at the center, we used pixels that included the additional printed silver mirror to reflect more than 90% of the IR radiation on the outer part of the pixel, thereby maintaining the temperature gradient within the TE legs. The data clearly proves the possibility of identifying the fingertip's position, both laterally and vertically.

**FIGURE 7:**
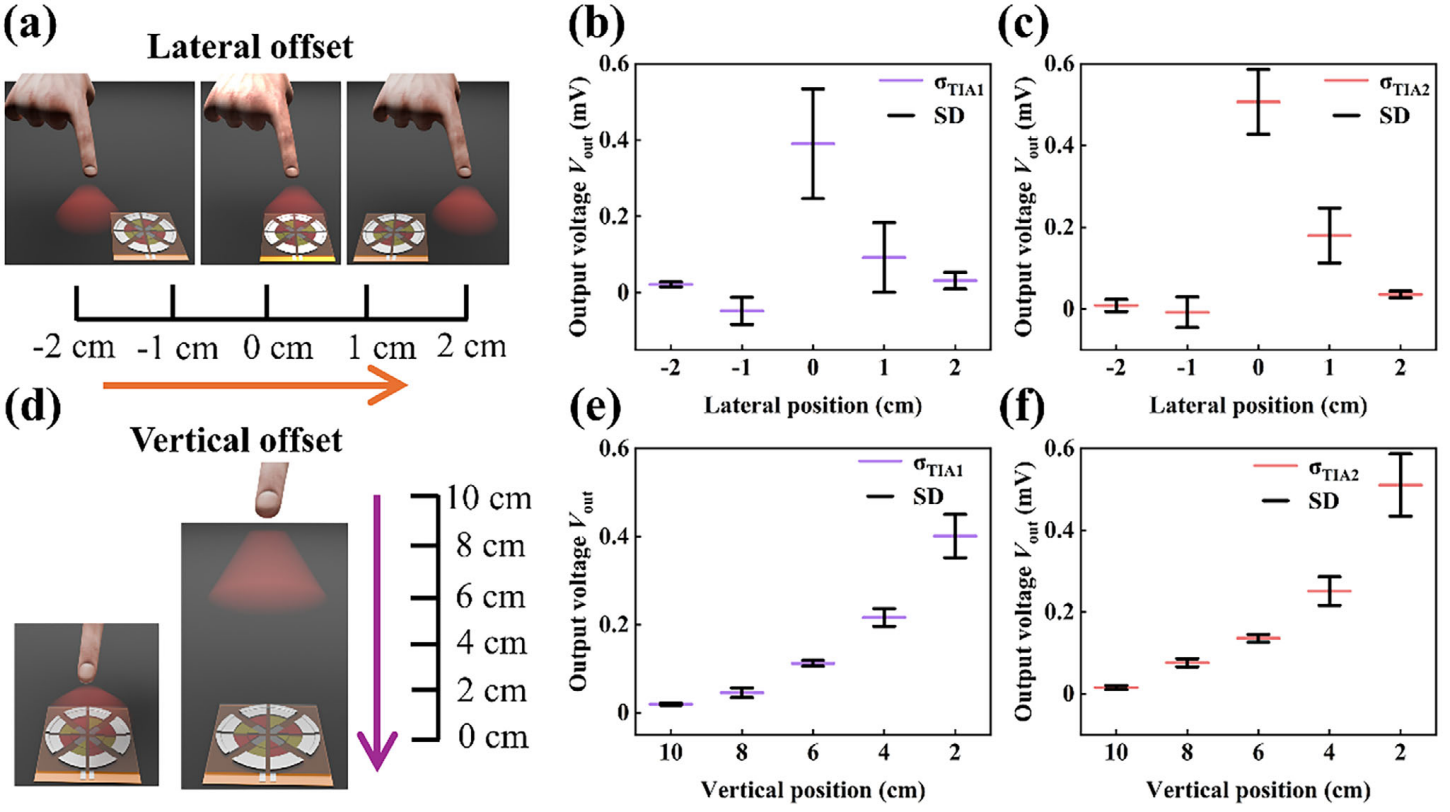
The effect of directional offsets on the response of the pixel. (a) Illustration of the discrete movement of the fingertip to imitate a lateral offset. (b) Response from TIA1 with different lateral positions. (c) Response from TIA2 with different lateral positions. (d) Illustration of the discrete movement of the fingertip to imitate a vertical offset. (e) Response from TIA1 with different vertical positions. (f) Response from TIA2 with different vertical positions.

### 3.5 Performance of TIAs for Contact‐Free HMI Applications

Following the characterization of individual pixels, the TIAs were integrated with customized readout electronics to demonstrate their use as HMI interfaces (cf. Figure 8a). The HMI systems comprise three components: TIAs, readout PCB, and the computer, with which we can visualize the responses from active and idle pixels. The overall function of the readout PCB is shown as a flow chart in Figure 8b, and the details of the engineered response speed, threshold setting, and overall latency are described in the Supporting Information (cf. Note S5). The 10‐pixel TIA1 was demonstrated as a contact‐free dialing pad (cf. Figure 8c), whereas the 16‐pixel TIA2 was demonstrated as a contact‐free writing screen (cf. Figure 8d). The idle‐state responses of every pixel within one multiplexing cycle are shown, and the gray dashed line represents the digital threshold that we defined accordingly. In the following demonstration, only the channels with responses that exceed it were recorded to accelerate the readout and signal processing. Other than that, the uniformity of output responses on different pixels was tested under a broader illumination source, with a pixel‐to‐pixel variation with in ± 10% (cf. Note S6).

**FIGURE 8:**
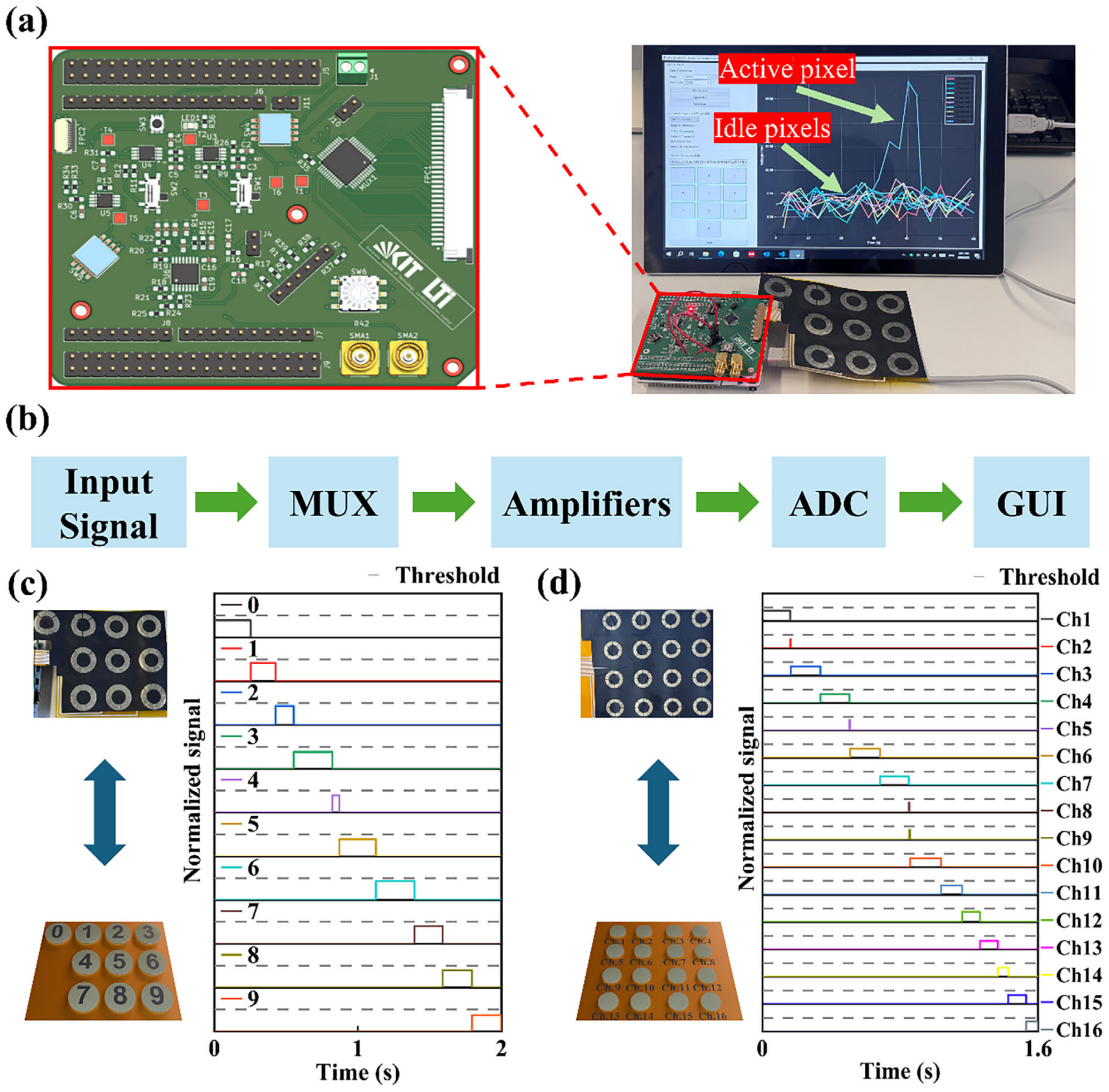
(a) Readout electronics and signal‐processing setup connected to a TIA, and an enlarged schematic view of the PCB. (b) Flowchart of the signal chain on the readout PCB. (c) Mapping of the contactless dial pad and (d) mapping of the contactless handwriting screen, with the idle states of time‐resolved responses of different pixels without any thermal stimulus. The signals therefore just represent noise. Hence, we defined the threshold accordingly.

#### 3.5.1 Demonstration of TIA1 as a Contact‐Free Dialing Pad

TIA1 was used to demonstrate a dialing pad. The output analog signals from the thermally stimulated pixels were converted into digital information: ten sensor pixels represent the decimal digits 0 to 9 (cf. Figure 8c). When the fingertip comes close to a pixel, the Seebeck voltage is generated via the infrared PTE effect, thereby activating the pixel from its idle state to a value above threshold. The multiplexer sampling interval was set to 200 ms for clearer visualization. A threshold voltage is defined in the Python script during signal processing to determine when a digit is registered on the GUI. At a 200‐ms sampling interval, a full multiplexing cycle across all pixels takes ∼2 s. In addition, preliminary demonstrations with a longer sampling interval are presented in Note S7.

Figure 9a,b shows examples of ascending dialing 1‐3‐5‐7‐9 (cf. Video S1) and descending dialing 8‐6‐4‐2, respectively. Furthermore, we conducted the continuous repeated‐dialing (0‐0‐0) demonstration (cf. Figure 9c) by activating pixel ‘0’ three times consecutively.

**FIGURE 9:**
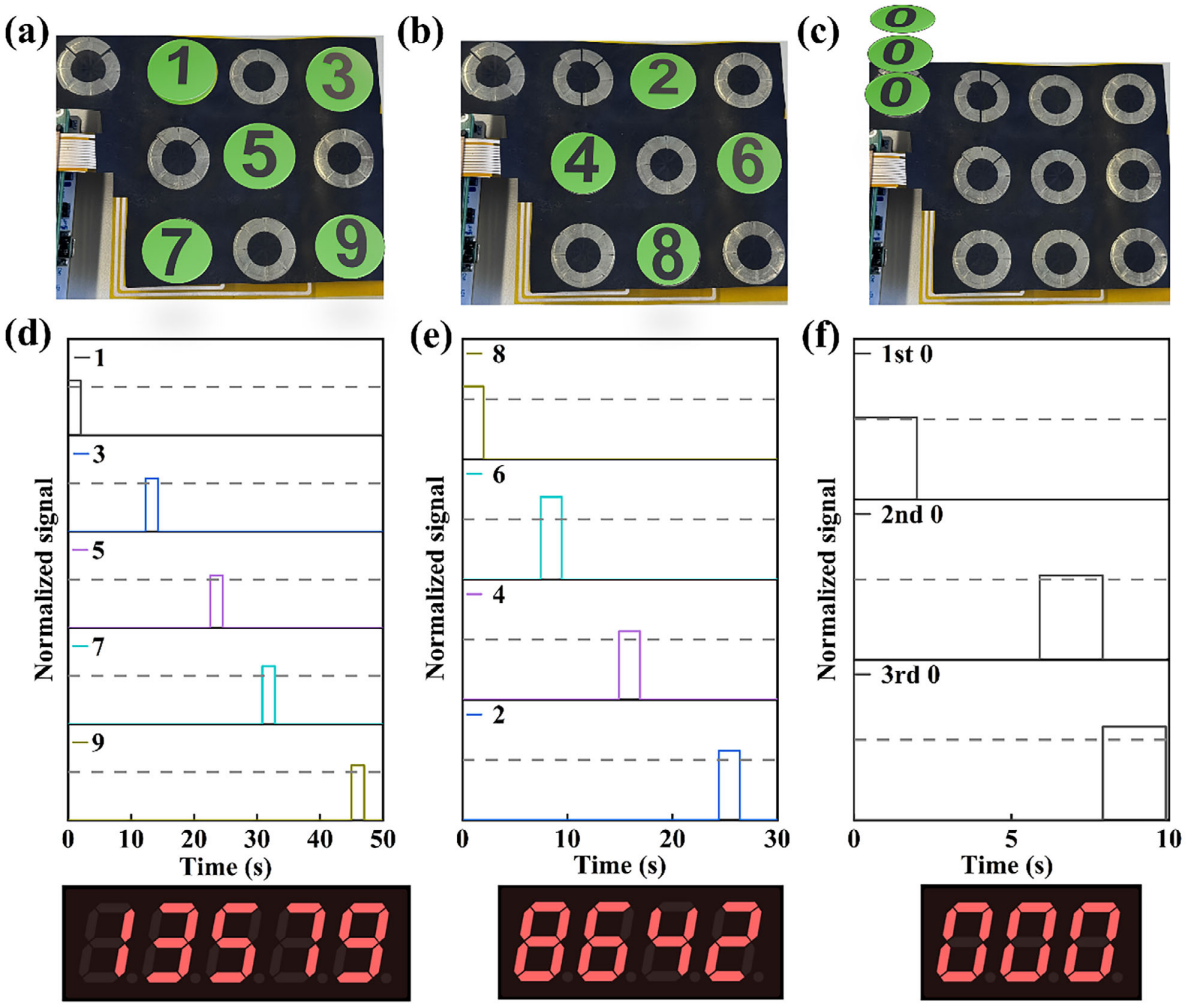
Demonstration of TIA 1 as a dialing pad. (a) Ascending dialing of 1‐3‐5‐7‐9. (b) Descending dialing of 8‐6‐4‐2. (c) Continuous dialing of 0‐0‐0. (d–f) The relevant pixels’ responses to the corresponding dialing.

#### 3.5.2 Demonstration of TIA2 as a Contact‐Free Writing Screen

A higher‐pixel‐density TIA2 was demonstrated as a contact‐free writing screen. Here, the sampling interval was set to 100 ms to achieve a faster scanning loop (∼1.6 s). Similarly, a threshold was set to select the pixels that were activated by a moving finger relative to their idle states (cf. Figure 8d). Figure 8d also shows a schematic of the mapping relationship between the TIA2 pixels and the digital array in the GUI. In this case, the pixel output voltages are normalized to their respective background noise levels. First of all, the letter ‘L’ is drawn on the writing screen, where, according to the mapping, the pixels 4‐8‐12‐13‐14‐15‐16 were sequentially activated, as shown in Figure 10a. Then we moved forward to more complicated shapes, which generally require higher resolution. This requirement fits quite well with the 4 × 4 sensing array. Figure 10b presents the writing of a right arrow (cf. Video S2), where the movement trajectory of the finger, as well as the corresponding output voltages, are shown in the diagrams.

**FIGURE 10:**
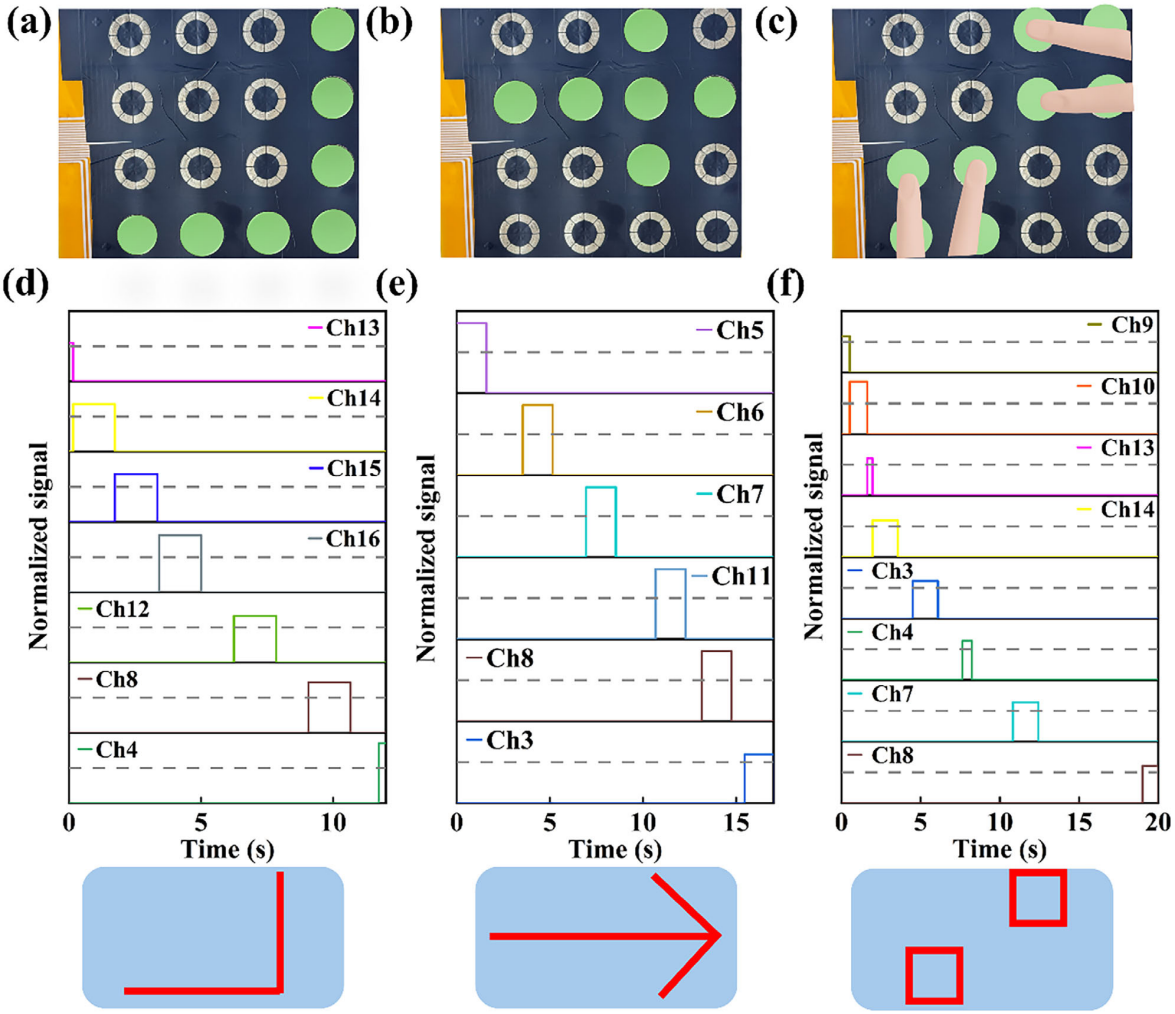
Demonstration of TIA 2 as a writing screen. (a) Drawing of the letter ‘L’. (b) Drawing of a right arrow. (c) Regional detection by four‐finger stimuli. (d–f) The relevant pixels’ responses of the corresponding drawing.

The same arrays can also be used as proximity sensors to locate a radiation source across different regions. To verify this function, we aim to detect two square regions where pixels are activated by fingers, as shown in Figure 10c. Here, two fingers were used to simulate large‐area IR sources at different positions in this demonstration. Despite the moderate speed to reach above threshold, the sensing precision of the localized thermal stimuli indicates that this TIA2 could be a promising candidate for proximity sensors. Statistics of success rate on a few different patterns performed on TIA1 and TIA2 are recorded in Note S8.

## 4 Simulation Results and Discussion

The finite element method (FEM) implemented in COMSOL Multiphysics was employed to compute the sensor response and to verify the experimental findings. In this study, two pixels with diameters of 20 and 15 mm were selected for simulation from each of the screen‐printed sensor arrays (TIA1 and TIA2, respectively). The corresponding 3D model of the pixel, along with the substrate, was implemented in COMSOL Multiphysics 6.3 for this purpose. In the 3D model, the camera view scale in the z‐direction is magnified by a factor of 50 to better visualize the thin printed layers (cf. Figure 11a – 15 mm pixel – TIA2). COMSOL Multiphysics was selected for this simulation because of its ability to handle coupled multi‐physics phenomena. In the software, material properties were assigned to all components of the modeled pixel: Kapton sheet as the substrate, silver (Ag) as the electrodes, carbon paste as an interface layer between the electrodes and the TE material, and n‐type BT and p‐type BST‐based films as the TE legs. Structural parameters of the pixel used in COMSOL are provided in Table S4. The physical modules, including heat transfer in solids and electric currents, were enabled for the relevant model domains. In the heat transfer module, an inward heat flux, defined by a Gaussian function from a 10‐mW laser, was applied to the center/absorber of the pixel. Surface‐to‐ambient radiation was enabled for all surfaces, with appropriate emissivities for each material (0.8 for the TE material and 0.75 for the Kapton substrate, based on experimental measurements). Convection heat transfer was also applied to all surfaces, assuming a convection heat transfer coefficient of ∼3 W m^−2^ K^−1^. After establishing the thermal boundary conditions, electrical boundary conditions were applied to the corresponding terminals – one set to floating potential and the other to ground. In Multiphysics module, electromagnetic heating was enabled for all current‐carrying domains, and the thermoelectric effect was only applied to the n‐type and p‐type TE domains. For meshing, a structured mesh type with an extremely fine element size was used to ensure simulation accuracy. Figure 11a shows the simulated 3D model of the reported sensor. Figure 11b,c demonstrates the simulated temperature distribution and electric potential for the TIA2 pixel, and Figure 11d represents the open‐circuit voltages (*V*_out_) under different laser powers for both TIA1 and TIA2 pixels. The potential and temperature profiles for the TIA1 pixel are presented in Figure S12. The slope of this *V*_out_‐*P*_in_ (V W^−1^) indicates the pixel's responsivity. Figure 11e,f shows the transient response of the pixels for TIA1 and TIA2, respectively. For the transient response, a laser power of 6 mW was applied for 20 s, then reduced to zero to simulate the signal's rise and fall. Good agreement with the experimental data is found (cf. Figure 6e,g). Minor differences can be explained by the fact that the real device exhibits imperfections due to printing roughness, porosity, and manufacturing errors that the model does not consider. In COMSOL, perfect contact between layers is assumed.

**FIGURE 11:**
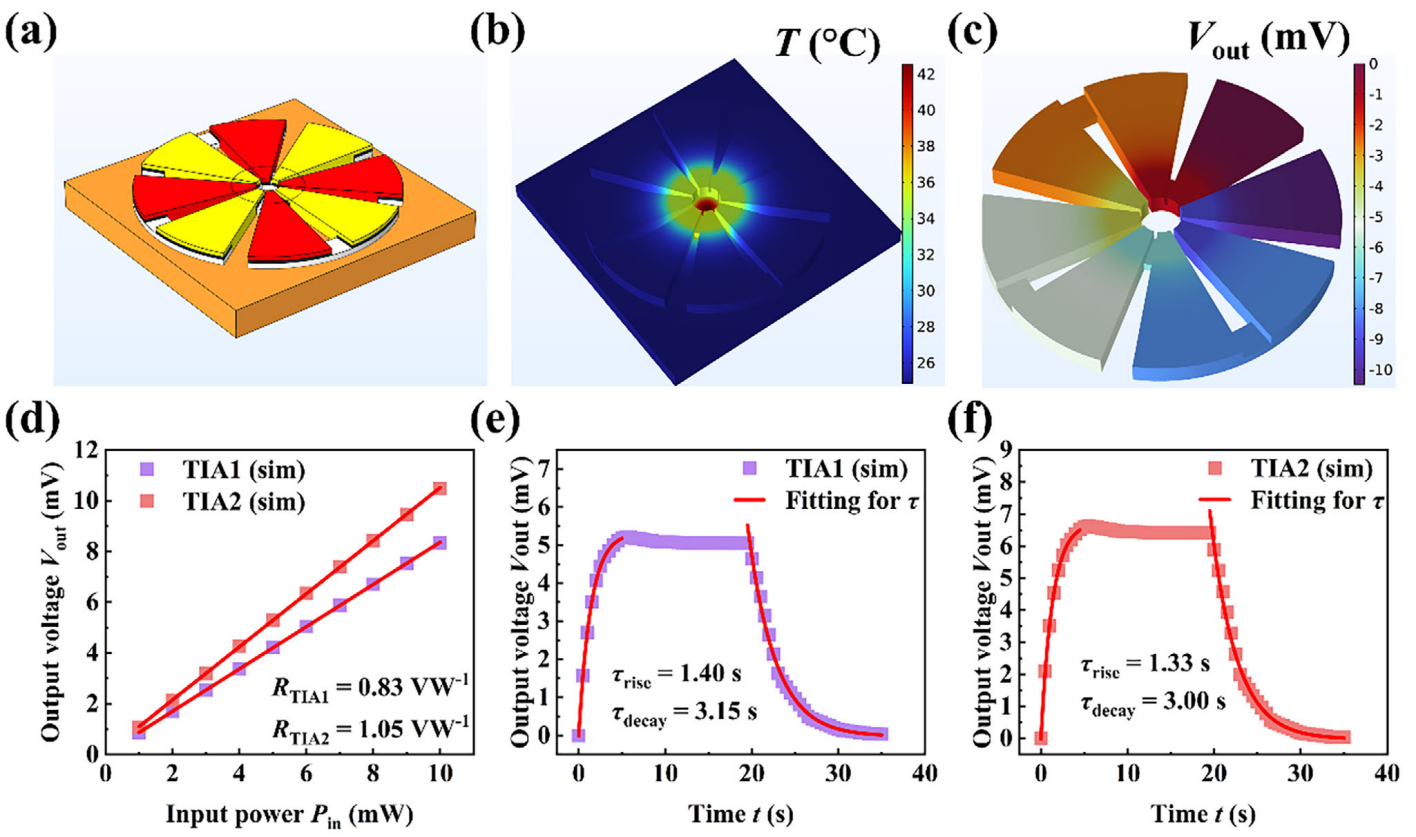
(a) 3D model of a TIA2 pixel. (b) Temperature distribution. (c) Electric potential distribution. (d) Pixels’ responsivity (V W^−1^), *V*_out_ vs. *P*_in._ (e) TIA1 pixel transient response, *V*_out_ vs. *t*. (f) TIA2 pixel transient response, *V*_out_ vs. *t*.

The temperature maps (cf. Figure 11b and Note S9) of both pixels show localized heating, with the cold junctions positioned beyond the estimated thermal healing length (*L_H_*), helping preserve the Δ*T*. The *L_H_* characterizes the in‐plane distance over which a localized temperature rise decays through heat conduction to the surroundings. For a printed film on a substrate with uniform in‐plane thermal conductivities (*k_TE_*, *k_sub_*) and thicknesses (*d_TE_*, *d_sub_*), the *L_H_* is defined as;

(3) $$L_{H}=\sqrt{\frac{k_{\mathrm{TE}}d_{\mathrm{TE}}+k_{\mathrm{sub}}d_{\mathrm{sub}}}{g}}$$

where $g=h_{\mathrm{top}}+h_{\mathrm{bottom}}+4\sigma T_{0}^{3}\left(\varepsilon_{\mathrm{top}}+\varepsilon_{\mathrm{bottom}}\right)$ is the heat‐loss co‐efficient per unit area. *T*_0_ is the ambient temperature and σ is the Stefan‐Boltzmann constant. Using the approximated values of *h* and ε, the *g* can be estimated to be 15 Wm^−2^K^−1^. Hence, *L_H_* is expected to be 1 mm for both pixels considering an average *k_TE_* of 0.5 Wm^−1^K^−1^ [8]. However, effective Δ*T* depend not only on dimension of a pixel but also on heat‐loss area (*A_loss_*).

(4) $$\Delta T=\frac{P_{\mathrm{in}}}{G_{\mathrm{TE}}+gA_{\mathrm{loss}}}$$

Shorter pixel (TIA2) would increase hot‐to‐cold conduction (*G_TE_*) if its width stayed constant. However, narrower radial pixel legs proportionally reduces width keeping *G_TE_* approximately unchanged. On the other hand, *A_loss_* decreases by more than 40%, consequently enhancing Δ*T*. Hence, higher responsivity of TIA2 may arise from a more favorable balance between lateral heat spreading and heat loss to the surroundings.

## 5 Conclusions

In summary, this study demonstrates flexible contact‐free infrared interface arrays (TIAs) fabricated using a combination of screen printing and aerosol jet printing. The printed TIAs detect thermal stimuli under long‐wave infrared illumination by leveraging the photo‐thermoelectric effect of Bi‐Te‐based printed thermocouples. With a pixel responsivity > 0.7 VW^−1^, the TIAs combine large‐area printability, flexibility, and spatially resolved contactless detection on a scalable platform. Beyond sensing performance, the TIAs were further demonstrated as dialing and handwriting interfaces, highlighting the practical usability for gesture‐based communication. These findings pave the way for printed thermoelectric infrared interface systems as a potential approach to self‐powered and shape‐conformable next‐generation human‐machine interfaces.

## Author Contributions

**Zirui Wang**: conceptualization, investigation, Writing – original draft, methodology, validation, visualization, Writing – review and editing, data curation. **Nan Luo**: writing – original draft. **Amrutha Bose**: writing – original draft. **Muhammad Irfan Khan**: writing – original draft, visualization, investigation. **Jiayi Liu**: methodology. **Leon Viktor Klinder**: validation, data curation. **Leonard Franke**: data curation, validation. **Mohamed Hussein**: investigation, visualization, validation. **Uli Lemmer**: writing – original draft, funding acquisition, writing – review and editing, project administration, resources. **Md Mofasser Mallick**: conceptualization, investigation, writing – original draft, writing – review and editing, methodology, supervision, project administration.

## Conflicts of Interest

The authors declare no competing interests

## Supporting information




**Supporting File 1**: advs78569‐sup‐0001‐SuppMat.docx.

**Supporting File 2**: advs78569‐sup‐0002‐VideoS1.mp4.

**Supporting File 3**: advs78569‐sup‐0003‐VideoS2.mp4.

## Data Availability

The data that support the findings of this study are available from the corresponding author upon reasonable request.
