# Supplementary material for: Contact‐Free Human‐Machine Interfaces via Printed Thermopile Infrared Arrays
Source: Adv Sci (Weinh). 2026 Oct 29:e78569. Online ahead of print. doi: 10.1002/advs.78569 (PMC13720272; doi:10.1002/advs.78569)
Supplement: Supplementary file 1 [file ADVS-9999-e78569-s001.docx]

**Supplementary Information**

**Contact-free human-machine interfaces via printed thermopile infrared arrays**

*Zirui Wang^1^, Nan Luo^1^, Amrutha Bose^2^, Muhammad Irfan Khan^1^, Jiayi Liu^1^, Leon Viktor Klinder^1^, Leonard Franke^1,3^, Mohamed Hussein^1,4^, Uli Lemmer^1,2 *^ and Md Mofasser Mallick^1,3,*^*

^1^Light Technology Institute, Karlsruhe Institute of Technology (KIT), 76131 Karlsruhe, Germany.

^2^Institute of Microstructure Technology, Karlsruhe Institute of Technology (KIT), 76344 Eggenstein-Leopoldshafen, Germany

^3^varmo GmbH, 76433 Eggenstein-Leopoldshafen, Germany

^4^Department of Physics, Faculty of Science, Ain Shams University, Abbassia, Cairo 11566, Egypt

*Corresponding authors: uli.lemmer@kit.edu; mofasser.mallick@kit.edu

**Table of contents**

Supplementary Table S1. Comparison table among different HMI systems

Supplementary Table S2. Dynamic viscosities of prepared TE ink.

Supplementary Fig. S1. Photographs of the different layers of TIA1 and TIA2. (a) Silver circuit of TIA2. (b) Carbon interfaces of TIA2. (c) p- and n-types of printed TE materials of TIA2. (d) Black carbon encapsulation tape layer of TIA2. (e) Silver circuit of TIA1. (f) Carbon interfaces of TIA1. (g) p- and n-types of printed TE materials of TIA1. (h) Black carbon encapsulation tape layer of TIA1.

Supplementary Fig. S2. Images of a screen-printed (SP) silver circuit under a light microscope. (a) A wide SP silver line at low magnification. (b)

Supplementary Fig. S3. SEM images of the AJP silver mirror. (a-c) Micro-cracks on the surface. (d) Cracks and some nanovoids, that are not covered by the nanoparticle silver ink. (at 3000 x magnification). (e)(f) Nanovoids and a crack at 15,000 x magnification.

Supplementary Fig. S4. White-light interferometry analysis of TIA1. The results include a 2D roughness distribution, a 3D topography map, and the linear roughness distributions along x and y.

**Note S1: Spatial and temperature resolution**

Supplementary Fig. S5. Spatial resolution of neighboring pixels. (a) Photoresponses of TIA1 pixels in a column. (b) Photoresponses of TIA1 pixels in a row. (c) Photoresponses of TIA2 pixels in a column. (d) Photoresponses of TIA2 pixels in a row. (e) Heatmap of the array while activating the central pixel.

**Note S2: Long-term and bending stability tests**

Supplementary Fig. S6. Output voltages of representative pixels after aging, bending, and under bending. (a) Normal TIA1. (b) Normal TIA2. (c) Aged TIAs following 100’ bending fatigue. (d) Aged TIAs under bending condition. (e) The setup used to measure bent TIAs.

**Note S3: Thermal noise characterization**

Supplementary Fig. S7. Electrical characterization of TIAs. (a) Zero-input state Johnson noise measurement. (b) Recorded *V*noise

**Note S4: The effect of position offsets from fingertip on the output of the sensing pixels**

Supplementary Fig. S8. The effect of directional offsets on the pixel response. (a) Illustration of discrete fingertip movement to simulate a lateral offset. (b) Response from TIA1 at different lateral positions. (c) Response from TIA2 at different lateral positions. (d) Illustration of discrete fingertip movement to simulate a vertical offset. (e) Response from TIA1 at different vertical positions. (f) Response from TIA2 at different vertical positions.

**Note S5: Readout electronics development**

Supplementary Fig. S9. Readout electronics development. (a) The preliminary readout setup with different components (MUX, ADC, amplifiers) on different PCBs, and the enlarged view of the multiplexer and the 30-pin FPC connector. (b) Highly integrated readout electronics connected to a PC GUI for visualization of active pixels. (c) Flowchart of the signal chain on the readout PCB.

**Note S6: Variation of different pixels’ responses**

Supplementary Fig. S10. Distribution of output voltages across pixels under broader illumination. (a) Vi / Mean(Vi) of TIA1 and their standard deviation. (b) Vi / Mean(Vi) of TIA2 and their standard deviation.

**Note S7: Preliminary demonstration of dialing pad with longer sampling interval**

Supplementary Fig. S11. Preliminary results of the non-contact dialing pad (TIA1) with a dwell time of 2 s per channel. The output signal was recorded by a computer and converted to a binary signal during post-processing. (a) Ascending dialing of 1-3-5-7-9. (b) Descending dialing of 8-6-4-2.

**Note S8: Success rates on different patterns**

Supplementary Table 3. Success rate statistics

Supplementary Table S4. Structural parameters of the IR sensor (COMSOL model).

**Note S9: The comparison between simulations from TIA1 and TIA2**

Supplementary Fig. S	12. COMSOL simulation results for a single pixel from TIA1 c. Compared with TIA2, we observe a smaller temperature gradient and a lower pixel potential. (a) Electric potential distribution. (b) Temperature distribution from the back side of the pixel. (c) Temperature distribution from the top side of the pixel.

Supplementary Table S1. Comparison table among different HMI systems

Supplementary Table S2. Dynamic viscosities of prepared TE ink.

| **Sample Name** | **Dyn. Visc.** | **Speed** | **Torque** | **Temperature** | **Shear Rate** | **Shear Stress** |
| --- | --- | --- | --- | --- | --- | --- |
|  | cP | rpm | % | °C | 1/s | N/m² |
| p-BST | 430.1 | 20 | 92.6 | 22 | 39.96 | 17.19 |
| n-BT | 896.5 | 10 | 96.5 | 22 | 19.98 | 17.91 |

Supplementary Fig. S1. Photographs of the different layers of TIA1 and TIA2. (a) Silver circuit of TIA2. (b) Carbon interfaces of TIA2. (c) p- and n-types of printed TE materials of TIA2. (d) Black carbon encapsulation tape layer of TIA2. (e) Silver circuit of TIA1. (f) Carbon interfaces of TIA1. (g) p- and n-types of printed TE materials of TIA1. (h) Black carbon encapsulation tape layer of TIA1.

Supplementary Fig. S2. Images of a screen-printed (SP) silver circuit under a light microscope. (a) A wide SP silver line at low magnification. (b) SP silver circuit lines were examined under high magnification. The line width and spacing were measured to be approximately 600 µm and 400 µm.

Supplementary Fig. S3. SEM images of the AJP silver mirror. (a-c) Micro-cracks on the surface. (d) Cracks and some nanovoids, that are not covered by the nanoparticle silver ink. (at 3000 x magnification). (e)(f) Nanovoids and a crack at 15,000 x magnification.

Supplementary Fig. S4. White-light interferometry analysis of TIA1. The results include a 2D roughness distribution, a 3D topography map, and the linear roughness distributions along x and y.

**Note S1: Spatial and temperature resolution**

Supplementary Fig. S5. Spatial resolution of neighboring pixels. (a) Photoresponses of TIA1 pixels in a column. (b) Photoresponses of TIA1 pixels in a row. (c) Photoresponses of TIA2 pixels in a column. (d) Photoresponses of TIA2 pixels in a row. (e) Heatmap of the array while activating the central pixel.

Here, the background noise levels of all three tested pixels were noted in every case. Afterwards, a threshold voltage was set higher than the background noise of all pixels. Subsequently, the central pixel was activated using a fingertip, and its photoresponses along with the surrounding pixels were recorded. Finally, all the data points were normalized to the threshold voltage (shown as “1” in Figure S10 (a-d)). The responses from neighbouring pixels are much lower than the threshold, which means false activation doesn’t happen due to the improper design of the geometry. Supplementary Fig. S5 (e) presents the simulation results, where the heatmap is shown when the central pixel is activated. A dominant temperature gradient exists within the central pixel. For the surrounding pixels, there is no significant thermal leakage. These support the experimental results that our current spacing are sufficient to differentiate from pixel to pixel.

We also supplemented the study with noise equivalent temperature difference (NETD) to better understand the temperature resolution of our interfaces. NETD can be expressed as

$$NETD=\Delta T \times V_{n}\div V_{s}$$

Where $\Delta T$ is the temperature difference between target and reference, $V_{n}$ is the RMS noise voltage, and $V_{s}$is the peak signal voltage. In the experiment, we measured the absorber’s temperature and reflector’s temperature using a non-contact temperature sensor, and their subtraction is estimated as $\Delta T$. With a temperature difference of 0.3 °C (0.6 °C) for TIA1 (TIA2) and the RMS noise voltages of 3 μV during experiments, the NETD is calculated to be 182.7 mK for TIA1 (136.7 mK for TIA2).

**Note S2: Long-term and bending stability tests**

Supplementary Fig. S6. Output voltages of representative pixels after aging, bending, and under bending. (a) Normal TIA1. (b) Normal TIA2. (c) Aged TIAs following 100’ bending fatigue. (d) Aged TIAs under bending conditions. (e) The setup used to measure bent TIAs.

Laser measurements on old pixels (stored under normal conditions for more than 6 months) after 100 bending cycles (10 mm bending diameter) of both TIAs were conducted, which can serve as references for performance degradation due to air oxidation. Compared with the normal performances of TIAs (c.f. Supplementary Fig. S6 (a)-(b)), the responsivities for TIA1 and TIA2 after aging and bending (c.f. Supplementary Fig. S6(c)) degrade only 4.2 % and 5.6 %, respectively. These results can prove that our printed TE materials and pixels have relatively good long-term stability and bending stability.

Furthermore, we also performed the laser test when the TIAs were bent, the setup of which is shown in Supplementary Fig. S6 (e). We have added output voltages vs. input power under a certain bending condition (~ 20 mm diameter) on representative pixels for both TIAs, as shown in Supplementary Fig. S6 (d). The output voltages and responsivities of TIAs are decreased by 7.5% and 4.7% compared with the last-step performance (c.f. Supplementary Fig. S6(c)). Regarding the noise level, we found that the zero-state voltage of the bent pixel is 0.03~0.04 mV, while the aged reference shows 0.002~0.004 mV. This indicates that bending will increase the electrical resistance and the noise voltage.

**Note S3: Thermal noise characterization**

Supplementary Fig. S7. Electrical characterization of TIAs. (a) Zero-input state Johnson noise measurement. (b) Recorded *V*_noise_

To characterize the internal noise level of a single sensing pixel, we measured its zero-input response and recorded 1000 readings. Then, the root-mean-square (RMS) voltage was calculated and displayed on the nanovoltmeter (Keithley 2182A). The TIA was placed inside the black cover to minimize the effect of visible light and was connected to the nanovoltmeter. The slow rate of reading indicated that the nanovoltmeter was operated at the number of power line cycles (NPLC) of 2.5 and a measuring bandwidth of 12 Hz. The measured noise level (standard deviation) was 18 nV, which is slightly higher than the calculated value (14 nV) due to additional flicker noise at low bandwidth.

**Note S4: The effect of position offsets from fingertip on the output of the sensing pixels**

Supplementary Fig. S8. The effect of directional offsets on the pixel response. (a) Illustration of discrete fingertip movement to simulate a lateral offset. (b) Response from TIA1 at different lateral positions. (c) Response from TIA2 at different lateral positions. (d) Illustration of discrete fingertip movement to simulate a vertical offset. (e) Response from TIA1 at different vertical positions. (f) Response from TIA2 at different vertical positions.

To investigate the effect of the lateral offset of the source, we segment the path of movement into 4 parts (1 cm for every segment) with the central point at 0 position (cf. Supplementary Fig. S8(a)). We stay at each position for 20 s to ensure we reach a stable output voltage. Supplementary Fig. S8(b)(c) shows the change in the averaged output voltage with respect to lateral positions when we try to change the lateral offset of the source to activate the TIA. At positions -2 and 2, due to the relatively long operating range, there is barely any output. When the source was kept at the -1 position, which is the inner edge of the silver mirror, partial energy was absorbed by the cold side of the TE leg. Initially, this drove the thermocouples toward a thermal equilibrium state, thereby causing a perturbation.

Finally, the equilibrium was reached, and the designed cold side (outer part) functioned as the hot side, which explains the small negative peak in the figure. At the 0 position, the pixel was normally activated, and the maximum output was reached within a short time. This also indicates a high speed of adaptive ability when a pixel was previously not properly activated. Interestingly, we found that after the IR source was moved to position 1, the output voltage also quickly dropped. However, it didn’t reach the negative value shown at the -1 position; instead, it settled at a positive value, suggesting a memory effect in the thermocouple. Furthermore, this memory effect mainly depends on how much heat was previously stored in the legs. At position 2, the output voltage returned to its initial state around zero. Overall, the pixel's performance across 5 different lateral offsets shows a highly directional response with minimal degradation. In our design, the spacing between pixels exceeds 2 cm, effectively eliminating the possibility of false activation.

Secondly, the effect of vertical offset was also investigated using the fingertip as the source. (cf. Supplementary Fig. S8 (d)). Similar to the investigation of lateral offset, 6 different positions (10, 8, 6, 4, and 2 cm) were determined along the vertical axis, at each of which the fingertip would remain for 20 s to stabilize the output voltage. Supplementary Fig. S8 (e) and (f) show that the relation between output voltage and vertical offset can be approximated by a quadratic curve. For thermal radiation emitted from a blackbody, the radiated power could be determined by Stefan-Boltzmann’s law: $P=A\sigma\varepsilon T^{4}$, where A is the area of the emitting surface, 𝜎 is the Stefan-Boltzmann constant, 𝜀 is the emissivity, and T is the temperature of the blackbody. The power received by the pixel, in our case, is inversely proportional to $r^{2}$, where $r$ is the distance between the fingertip and the pixel. This effect of vertical offset basically describes the property of finger radiation for an HMI application, where it is more advantageous to move the IR source closer to the sensor. In addition, men can easily determine the effective working range when background noise and body temperature are held constant.

**Note S5: Readout electronics development**

Supplementary Fig. S9. Readout electronics development. (a) The preliminary readout setup with different components (MUX, ADC, amplifiers) on different PCBs, and the enlarged view of the multiplexer and the 30-pin FPC connector. (b) Highly integrated readout electronics connected to a PC GUI for visualization of active pixels. (c) Flowchart of the signal chain on the readout PCB.

To achieve HMI demonstrations, one of the most important considerations is to capture the relatively small output signals (sub-mV level) from the TIAs. On the other hand, for the purpose of real-time and multi-pixel demonstration, it is necessary to separately obtain the signal from each single signal channel. Most significantly, we would like to keep the size of the overall device small and scalable. Therefore, the readout circuit was developed from multiple test PCBs with different components into one highly integrated PCB, as shown in Supplementary Fig. S9. From the TIAs' perspective, the left contacts of every pixel are connected in series, forming the mutual electrode, which will go to the ground of the readout PCB. The right contacts of every pixel are extended to work as the electrodes of input channels, which will feed the signal to the PCB via a zero-insertion-force connector. Next, the output signal reaches the multiplexer, which opens only one output channel for a fixed dwell time and sends the signal to the subsequent procedures. Afterward, multiple amplification stages with tunable gains will follow to amplify the data to a desired level. In the following demonstrations, two amplification stages were used with a fixed gain of 1000*. Eventually, the amplified signal channel would pass through an ADC with a reference voltage of 2.048 V and an internal gain of 1. After analog-to-digital conversion, the data will be transported to the PC via a serial port for post-processing. Ultimately, we will obtain a consecutive sequence of data points from different pixels, each separated by an identical dwell time.

In previous sections, the time constant of a single pixel was discussed, and it was on the order of a few seconds. However, with the support of electronics, the real response time can be reduced by setting a suitable sampling interval and threshold voltages. Faster sampling on every signal channel can boost readout speed because it is not necessary to reach the pixel's maximum output. Therefore, a sub-second response could be reached, given a relatively long intrinsic time constant of the pixels. The noise level at idle states for both TIAs is recorded within one multiplexing cycle and shown in Figures 8(c) and 8(d), where the grey rectangles represent the digital threshold for the pixels’ activation. The intrinsic time constant is when the transient response changes to approximately 63 % of the saturated value, while this value can be tuned through the readout circuit to enhance the response time. Theoretically, the time for a pixel to reach the detection threshold should be shorter than the intrinsic time constant, which represents the engineered response speed. However, there will be some trade-offs, such as parasitic electrical resistance caused by the readout circuit. Due to the increased resistance, the intrinsic noise level will be even higher and amplified by the readout. Therefore, a more delicate parametric design of the circuit should be utilized (e.g. reference voltage, sample per second, and sampling interval, and digital threshold).

The per-channel sampling interval is defined by the channel opening time of the multiplexer. For the threshold settings, we should consider a value between the background noise from every channel and the maximum response the channels can achieve. A lower threshold makes the pixel more sensitive and responds faster, but potentially results in a higher false trigger rate, and vice versa for a higher threshold value. Due to the variation of 10 % from pixel to pixel, the threshold value could also be varied in 10 % range for different pixels in the experiment.

The overall system latency may come from 3 parts: 1. The synchronization between finger movement and the multiplexer. This is because when the pixel is about to cross the threshold, the open channel of the multiplexer is not the pixel you want to activate, and one more cycle is required. 2. Overheating of the pixel. Sometimes the pixel already accumulates enough heat to cross the threshold, but we don’t realize it. 3. The delay in communication between the analog components and the PC. After running for a long time, the components are hotter, and the PC cannot receive the data instantly. Based on this, the typical delay is around 2-4 seconds to see the displayed result, depending on how high we set the threshold.

Regarding the reset procedure for repeated inputs, we regret that we didn’t have any elaborate methods to achieve that. In our demonstration, we just selected the output that first came above the threshold and ignored other repeated inputs afterwards. Then we wait until this channel is below the threshold and activate again.

**Note S6: Variation of different pixel’s responses**

Supplementary Fig. S10. Distribution of output voltages across pixels under broader illumination. (a) $\sigma_{i}$ of TIA1 and their standard deviation. (b) $\sigma_{i}$ of TIA2 and their standard deviation.

Here, we used a hot plate with a black coating set to 40 °C to mimic a good blackbody emitter. Next, we illuminated all the pixels homogeneously. In the meanwhile, we moved our arrays from a relatively long distance to closer distance and recorded all the output voltages $V_{i, j}$ (*i* means the pixel number and *j* is the repetition number). Suppose there are, in total, *m* pixels and *n* repetitions.

$$\sigma_{j}= \frac{\sum_{i=1}^{m} V_{i, j}}{m}$$

Firstly, the averages of all channels’ output voltages, $\sigma_{j}$, for every repetition are calculated. Next, we use the per-channel-repetition $V_{i, j}$ to divide by $\sigma_{j}$ to get the per-channel deviation per repetition.

$$D_{i, j}= \frac{V_{i, j}}{\sigma_{j}}$$

Finally, averaged per-channel deviations $\sigma_{i}$ are calculated, which can illustrate the uniformity of pixels for many repeated measurements.

$$\sigma_{i}= \frac{\sum_{j=1}^{n} D_{i, j}}{n}$$

Therefore, $\sigma_{i}$ with the SD are located between 90 % and 110 %, as shown in Supplementary Fig. S10. Two different arrays (TIA1 & TIA2) were tested, and more than 50 repeat multiplexing cycles were finished, representing 50 repeated measurements.

**Note S7: Preliminary demonstration of dialing pad with longer sampling interval**

Supplementary Fig. S11. Preliminary results of the non-contact dialing pad (TIA1) with a dwell time of 2 s per channel. The output signal was recorded by a computer and converted to a binary signal during post-processing. (a) Ascending dialing of 1-3-5-7-9. (b) Descending dialing of 8-6-4-2.

When we first tested the TIA1s, a dwell time of 2 s per channel was set, and 9 channels were included. A longer dwell time helps the experimenter better recognize the output signal and synchronize with the multiplexer. After all data were collected, the output voltages for each channel were manually converted to binary. Two modes of activation were demonstrated: ascending and descending dialing. In the ascending dialing mode, all the odd numbers’ information could be collected within one multiplexing cycle. However, the even numbers’ information, from large to small, had to be collected within four cycles. This also underscores the importance of a short dwell time, as shown in the main text.

**Note S8: Success rates on different patterns**

We have repeated different patterns 20 times for both the TIAs. Overall, the success rate is found to be higher than 80 % if every threshold is properly set. The failure can be attributed to either false trigger of other unwanted pixels activated by other fingers of hand or too much time taken.

| Supplementary Table S3. Success rate statistics | | |
| --- | --- | --- |
| Array configuration | Patterns | Success rate |
| TIA1 | 3-5-2-8 | 17/20 |
| TIA1 | 4-7-9-0 | 19/20 |
| TIA2 | L-shape | 17/20 |
| TIA2 | up-arrow | 16/20 |

| Supplementary Table S4. Structural parameters of the IR sensor (COMSOL model). | | |
| --- | --- | --- |
| **No. of Layers** | **Material** | **Thickness (µm)** |
| Layer 1 | Substrate (Kapton) | 25 |
| Layer 2 | Conductor (Ag ink) | 10 |
| Layer 3 | Diffusion barrier (Carbon ink) | 5 |
| Layer 4 | n-type (Bi_2_Te_3_) | 22 |
| Layer 5 | p-type (Bi_2_Te_3_) | 22 |

**Note S9: The comparison between simulations from TIA1 and TIA2**

Supplementary Fig. S	12. COMSOL simulation results for a single pixel from TIA1 c. Compared with TIA2, we observe a smaller temperature gradient and a lower pixel potential. (a) Electric potential distribution. (b) Temperature distribution from the back side of the pixel. (c) Temperature distribution from the top side of the pixel.

The effective temperature gradient is generated only over a short length called the thermal healing length, where vertical heat dissipation dominates in our case. The temperature gradient will only be generated within this thermal healing length. The extra length of the leg will increase the thermal mass of the whole leg, where some of the energy will be used to heat up the part outside the thermal healing length. By contrast, a longer TE leg (TIA1) produces a lower effective temperature gradient and a slower speed to reach equilibrium. Simulations sweeping leg length also confirm this effect, indicating that a shorter TE leg (TIA2) yields a higher output voltage, thus better responsivity and shorter time constants.
